# Case Report: Successful telitacicept treatment for IgA nephropathy with stage 4 chronic kidney disease and acute renal failure

**DOI:** 10.3389/fimmu.2026.1753920

**Published:** 2026-07-14

**Authors:** Fang Zeng, Fei Tan, Dehui Liu, Fang Wang

**Affiliations:** Department of Nephrology, Ganzhou Hospital-Nanfang Hospital, Southern Medical University (Ganzhou People’s Hospital), Ganzhou, China

**Keywords:** acuterenal failure, estimated glomerular filtration rate, IgA nephropathy, stage 4 chronic kidney disease, telitacicept

## Abstract

Immunoglobulin A nephropathy (IgAN) is one of the most common forms of primary glomerulonephritis and can lead to renal failure requiring renal replacement therapy via dialysis. Current research indicates that telitacicept, an APRIL and BAFF inhibitor, has shown promise as a therapeutic option for IgAN patients with an estimated glomerular filtration rate (eGFR) over 30 mL/min/1.73 m². However, the efficacy of telitacicept in slowing the deterioration of kidney function, especially in individuals with stage 4 chronic kidney disease (CKD) and advancing IgAN, has yet to be documented. We present a 47-year-old male with stage 4 CKD, complicated by acute kidney injury and diagnosed with IgAN via biopsy, who was initially treated with 240 mg of telitacicept for 6 months, then transitioned to a gradual reduction and maintenance therapy for 12 months. After treatment, the proteinuria decreased from 2.82g/day to 0.60g/day. The eGFR gradually increased, improving from 26.95 to 35.69 mL/min/1.73 m² over 18 months. Serum albumin levels increased steadily, while hematuria gradually declined. Immunoglobulins and lymphocytes exhibited a decreasing pattern during treatment, with no notable rise in inflammatory markers. Furthermore, no infections or other adverse events were observed. This case shows the potential for telitacicept to be applied beyond its approved indications. This emphasizes the importance of conducting more research on individuals with advanced chronic kidney disease.

## Introduction

Immunoglobulin A nephropathy (IgAN) is the most widespread primary glomerulonephritis around the world, carrying a high risk of enduring renal failure and a notable socioeconomic burden (1). IgAN is characterized by a wide range of clinical and pathological manifestations and involves a complex pathogenesis, in which B lymphocyte activation plays a pivotal role, particularly during the early stages of the disease. Early CD20^+^ B cells synthesize limited quantities of galactose-deficient IgA1 (Gd-IgA1), whereas CD38^+^ plasma cells produce both Gd-IgA1 and antibodies against Gd-IgA1 (2). These two components form immune complexes that accumulate in the glomerular mesangium, leading to mesangial proliferation, cytokine release, and complement activation, which subsequently induces inflammation and ultimately result in renal function decline (3, 4).

Telitacicept, a recombinant fusion protein, comprises the extracellular domain of the transmembrane activator and calcium-modulator and cyclophilin ligand interactor linked to the Fc region of human immunoglobulin G1 (5). Telitacicept exhibits a high affinity for binding to B lymphocyte stimulator and a proliferation-inducing ligand, thereby interfering with the development, maturation, and survival of B cells and plasma cells at various stages. This mechanism aids in controlling disease progression and mitigating damage to target organs. Empirical studies have demonstrated that telitacicept effectively reduces circulating levels of Gd-IgA1 and IgA-containing immune complexes, with these reductions correlating with a decrease in proteinuria (6). A phase II clinical trial of telitacicept was conducted involving patients with IgAN and an estimated glomerular filtration rate (eGFR) greater than 35 mL/min/1.73 m^2^ to assess its efficacy and safety (7). However, the efficacy and safety of this medication for patients with decreased eGFR remain insufficiently studied.

In this article, we present the first reported case of a patient with IgAN, stage 4 chronic kidney disease (CKD), and acute renal failure (ARF) treated with telitacicept, along with an 18-month follow-up.

## Case report

The patient was a 47-year-old male who presented to the Department of Nephrology, Ganzhou Hospital-Nanfang Hospital on April 20, 2024, with a chief complaint of “elevated serum creatinine detected for 2 days”. The patient reported a dull pain in the kidney region on April 18, 2024, with increased foamy urine and nocturia occurring 4–5 times nightly, but no urgency, frequent urination, or visible hematuria. The 24h urine volume was normal. He also complained of fatigue and nausea, without abdominal pain, edema, fever, chest tightness, skin rash, joint pain, dry mouth, or dry eyes. He sought care at a local hospital, where laboratory tests revealed the following: serum creatinine (Scr) 564.42 µmol/L; urinalysis showed proteinuria (2+) and urinary red blood cell (RBC) count (2+) on April 20. To further evaluate possible renal pathology, he was referred to our hospital. Throughout the illness, the patient experienced poor mental status, appetite, and sleep, while maintaining normal yellow stool consistency. He denied any history of kidney disease, hypertension, diabetes, or coronary artery disease. His personal and family histories were unremarkable.

Physical Examination upon Admission: Temperature: 36.0 °C; Pulse: 93 beats/min; Respiratory rate: 20 breaths/min; Blood pressure: 157/105 mmHg; BMI: 26.6 kg/m^2^. The skin showed no rash, purpura, or ulceration. There was no facial or lower extremity edema. Cardiovascular, pulmonary, and abdominal examinations revealed no abnormal positive findings. Laboratory Examinations: Complete Blood Count: White blood cell count: 7.3 × 10^9^/L; Hemoglobin: 102 g/L ↓; Platelet count: 331 × 10^9^/L. Renal function: Urea: 13.9 mmol/L ↑; Scr: 533.0 µmol/L ↑; eGFR: 13.2 mL/min/1.73 m² ↓; Uric acid: 698.1 µmol/L ↑; Parathyroid hormone: 129.9 pg/mL ↑. Hepatic function, blood lipids, blood glucose, cardiac enzymes, C-reactive protein, hepatitis B markers, pre-transfusion tests, female tumor markers, and thyroid function were all within normal limits. Immunologic Tests: antinuclear antibody panel, vasculitis screening, rheumatoid factor, immunoglobulin profile, complement levels, serum free light chains, and serum protein electrophoresis showed no significant abnormalities. Urinalysis: Proteinuria: 3+; RBC count: 19.5 cells/µL; white blood cells: 2/HPF; 24-hour urinary protein excretion: 2.8 g/day. Imaging tests: Renal and Renal Vascular Ultrasound: Right kidney: 10.4 cm × 5.0 cm×3.0 cm, cortical thickness 9 mm; Left kidney: 10.4 cm×4.9 cm×3.0 cm, cortical thickness 9 mm. Bilateral kidneys showed diffuse parenchymal changes, reduced intrarenal perfusion, and decreased blood flow velocity. Dynamic Renal Scintigraphy: Decreased perfusion in both kidneys; severely impaired renal function (GFR: left kidney 14.36 mL/min/1.73 m², right kidney 12.59 mL/min/1.73 m^2^).

**Figure 1:**
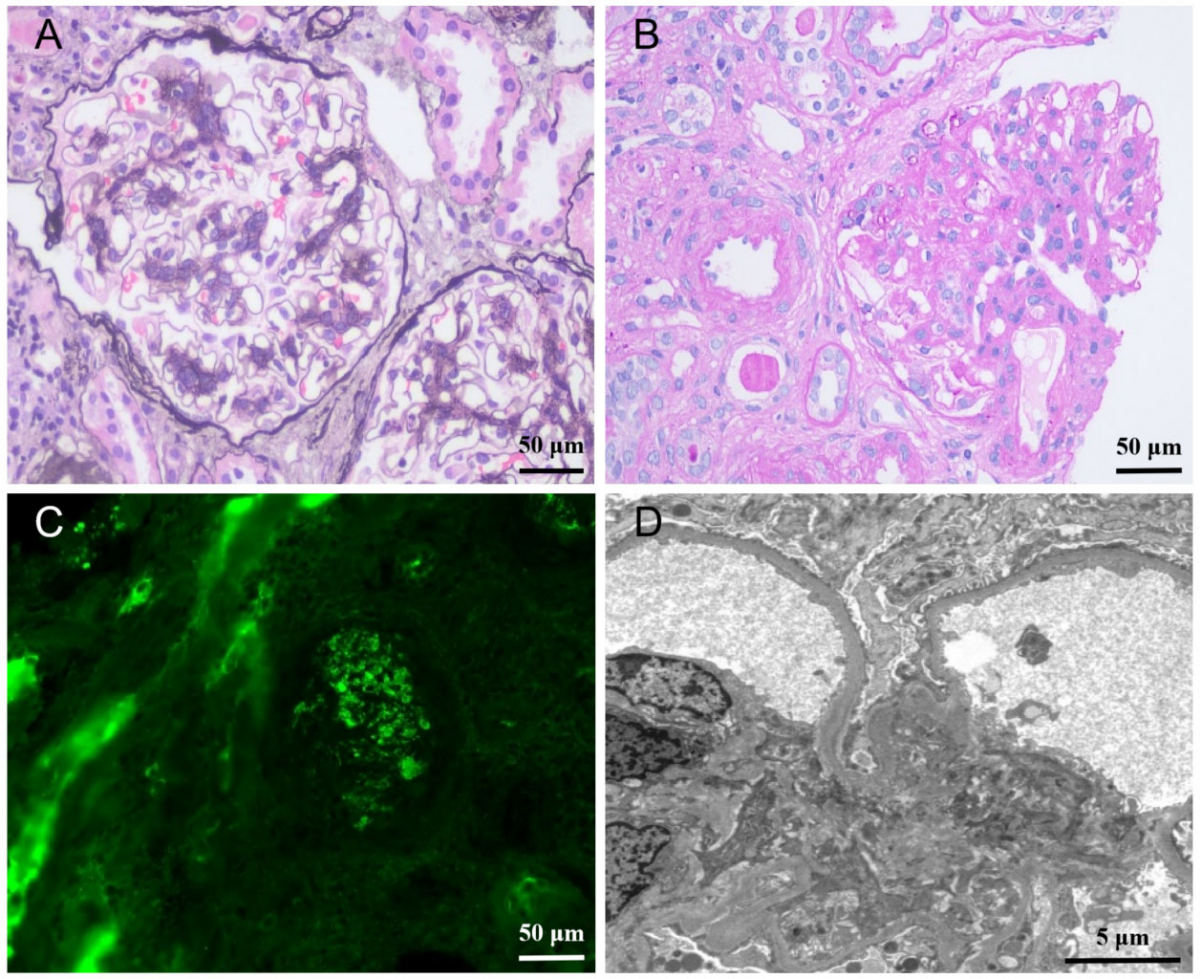
Pathological analysis of the renal biopsy tissue. **(A)** Mesangial cell hyperplasia (Periodic acid-Silver methenamine stain, ×400); **(B)** Increased mesangial matrix and mesangial cells (Periodic acid-Schiff stain, PAS, ×400); **(C)** Deposition of IgA immune complexes by immunofluorescence (Immunofluorescence microscopy, ×400); **(D)** Deposition of electron dense material in the mesangial region by electron microscopy.

Scr was notably high upon admission, along with increased proteinuria, hematuria, and blood pressure. The initial treatment included amlodipine besylate for blood pressure control, febuxostat for uric acid reduction, sodium bicarbonate tablets for urine alkalinization, coated aldehyde oxystarch capsules for intestinal dialysis, roxadustat for renal anemia management, and gastroprotective therapy. By the sixth day of medication, the patient’s symptoms of fatigue and nausea had not improved, and repeat testing showed a further rise in Scr to 552 µmol/L. According to the results, the diagnosis is CKD stage G4 with ARF. The patient consented to deep venous catheterization and underwent three sessions of hemodialysis. By the ninth day of hospitalization, the symptoms improved enough to discontinue dialysis. Dynamic monitoring revealed that Scr levels ranged from 265 to 285 µmol/L during days 10 to 16. To clarify the cause of ARF, a renal biopsy was performed on day 17. Light microscopy revealed 42 glomeruli: 21 showed global sclerosis and 10 showed segmental sclerosis; no crescents were observed. The remaining glomeruli exhibited moderate mesangial cell and matrix proliferation, with focal and segmental exacerbation. Tubular epithelial cells displayed vacuolar degeneration, protein casts were observed, and a few tubules showed luminal dilation with loss of brush border. Multifocal and patchy atrophy was noted, involving approximately 65% of the sampled tissue. The renal interstitium showed multifocal inflammatory cell infiltration with fibrosis. Small arteries had thickened walls, and arterioles demonstrated hyaline changes and narrowed lumens. Immunofluorescence revealed granular mesangial deposits of IgA (2+), IgM (1+), and C3 (2+). Electron microscopy examined seven glomeruli, with three showing global sclerosis and three segmental sclerosis. Capillary loops were open with segmentally thickened basement membranes (approximately 300–550 nm). Podocytes showed vacuolar degeneration with partial foot process effacement. Mesangial cell and matrix proliferation was present, with electron-dense deposits visible. The pathological diagnosis was IgAN, classified as Lee grade V and Oxford classification M1E0S1T2C0 (**Figure 1**). According to the diagnostic process of the 2025 Global Kidney Disease Prognosis Improvement Organization (KDIGO) guidelines (8), primary IgAN was clinically diagnosed after excluding secondary factors.

**Figure 2:**
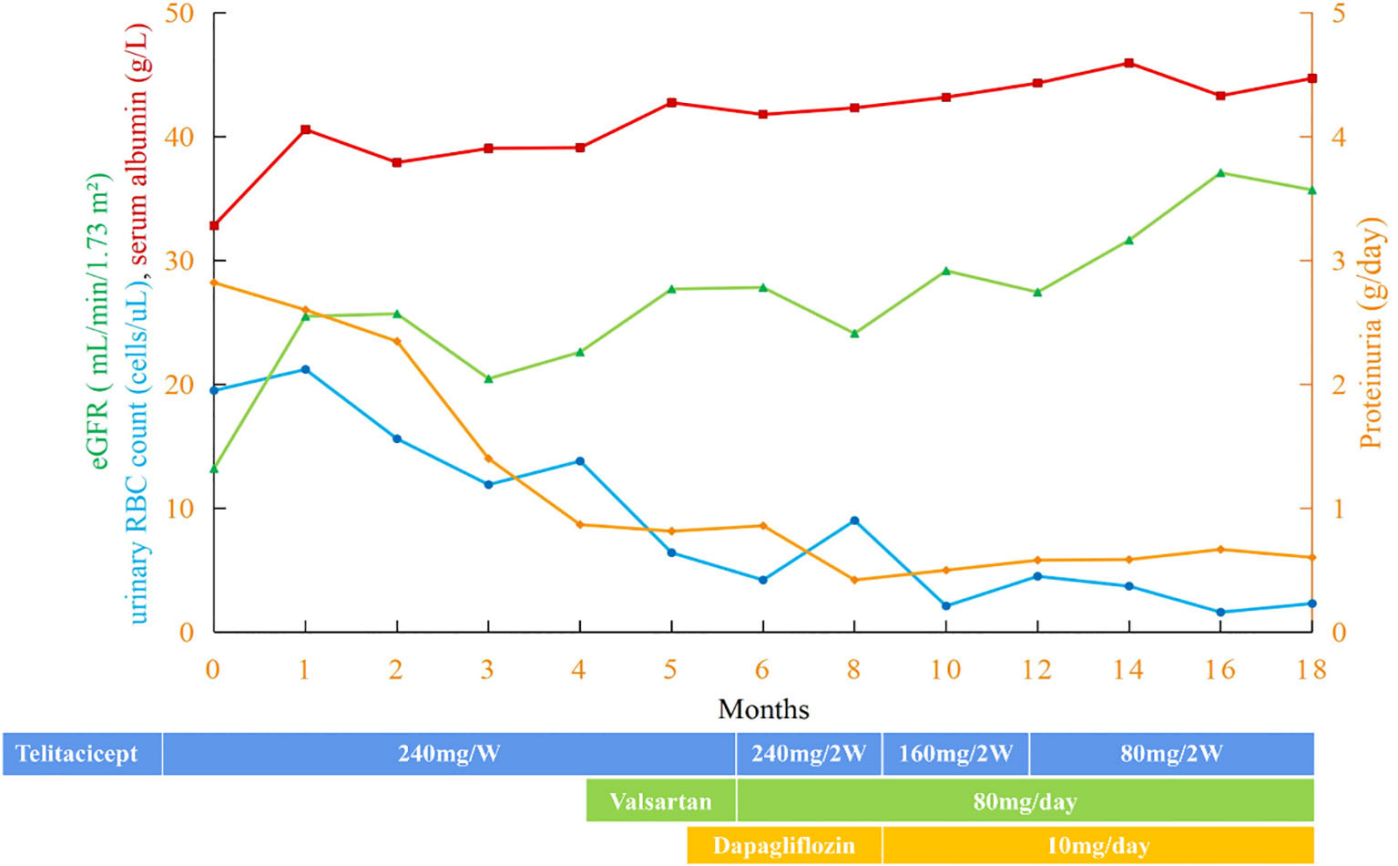
Changes of serum proteinuria, urinary RBC count, eGFR, and serum albumin over the 18-month follow-up period.

In light of the patient’s substantial concerns regarding the adverse effects of corticosteroid therapy-such as potential exacerbation of pre-existing hypertension, increased risk of renal osteodystrophy, heightened susceptibility to gastrointestinal mucosal injury, and an elevated risk of infection, which is already pronounced due to severe renal insufficiency—along with the safety profiles of immunosuppressive agents, particularly apprehensions about bone marrow suppression, life-threatening infections, nephrotoxicity, and the reproductive toxicity associated with alkylating agents, an alternative treatment approach was considered. Telitacicept (manufactured by Rong chang Biopharmaceutical Co., Ltd., 80 mg/vial), which effectively mitigates these adverse effects while targeting the underlying immune-mediated pathology of the disease, was initiated on May 8 at a dosage of 240 mg once weekly. The dosing regimen was subsequently adjusted to 240 mg every two weeks after six months, reduced to 160 mg every two weeks after nine months, and further decreased to 80 mg every two weeks after twelve months, which was then maintained. During the 18 months treatment period with telitacicept, the patient experienced a progressive reduction in proteinuria, while eGFR improved steadily. Trends in proteinuria, urinary RBC count, eGFR, and serum albumin are illustrated in **Figure 2**. The patient’s blood pressure was maintained at approximately 125/70 mmHg. When looking at further parameters (**Table 1**), We noted a gradual normalization of serum uric acid, a decrease in immunoglobulins and lymphocytes, and stable levels of hemoglobin, complement, and inflammatory indicators. Importantly, as of his most recent appointment in November 2025, the patient had not experienced any significant drug-related side effects, such as injection site reactions, infections, gastroenteritis, or anemia.

**Table 1:** Change in selected parameters over the treatment period.

Parameter	Baseline	3 months	6 months	9 months	12 months	15 months	18 months
White blood cell (×109/L)	7.30	6.97	5.67	6.15	6.60	9.43	5.83
Hemoglobin (g/L)	102	127	150	111	120	114	139
Serum uric acid (µmol/L)	698	476	444	387	416	361	325
CRP (mg/L)	4.21	1.75	2.38	0.96	2.07	1.18	1.72
Serum IgG (g/L)	11.15	8.24	6.57	7.08	6.82	7.17	7.02
Serum IgA (g/L)	1.75	1.31	0.75	0.84	0.92	0.97	0.89
Serum IgM (g/L)	1.47	1.01	0.88	0.83	0.96	0.81	0.85
Serum C3 (g/L)	0.99	1.18	0.76	0.70	0.82	0.88	0.81
Serum C4 (g/L)	0.25	0.32	0.21	0.21	0.22	0.25	0.23
CD4+ T cells (n/µL)	1110	807	899	1013	976	907	981
CD19+ B cells (n/µL)	394	196	232	208	259	187	214

CRP, C-reactive protein; IgG, Immunoglobulin G; IgA, Immunoglobulin A; IgM, Immunoglobulin M.

## Discussion

This case report illustrates the successful 18-month application of telitacicept in treating patients with advanced IgAN who had an eGFR lower than 30 mL/min/1.73 m^2^.

The pathophysiological mechanisms underlying IgAN remain inadequately understood. The disease usually has a chronic and progressive course, often presenting as asymptomatic hematuria and proteinuria, or as nephritic syndrome (9). Presently, specific pharmacological interventions for IgAN are lacking. In a cohort from the United Kingdom with biopsy-confirmed IgAN and proteinuria exceeding 0.5 g/day or an eGFR below 60 ml/min per 1.73 m^2^, it has been projected that nearly all patients will advance to end-stage renal disease (ESRD) within their lifetime, barring a reduction in eGFR decline to less than 1 ml/min per year (10). The progression rate may be more accelerated in Asian populations (11, 12).

Hypertension and proteinuria are pivotal in the progression of IgAN. According to the 2025 KDIGO guidelines (8), initial management of IgAN should be supportive, emphasizing intensive blood pressure control and adequate blockade of the renin-angiotensin-aldosterone system. Nonetheless, some patients may be unable to tolerate these medications due to reduced eGFR. In this instance, the patient was diagnosed with CKD stage G4, with limited options for supportive therapy. Renal pathology indicated the presence of chronic lesions with active components, accompanied by proteinuria and hypertension, both of which are recognized risk factors for renal function decline and progression to ESRD. Consequently, we evaluated the potential benefit of immunosuppressive therapy for the patient. However, the use of steroids in treating primary IgA nephropathy in high-risk patients with progressive disease remains controversial due to the numerous adverse effects associated with these medications. The TESTING study, the largest clinical trial in international glomerular disease research, suggests that full-dose corticosteroid therapy, as recommended by current clinical practice guidelines for IgA nephropathy, may significantly increase the risk of adverse events for patients (13). Moreover, the 10-year follow-up results of the notable STOP study confirmed that the addition of immunosuppressive agents to supportive therapy for patients with IgA nephropathy did not result in statistically significant differences in outcomes (14). Consequently, existing therapeutic strategies may not sufficiently address the progression of disease in these patients.

The current body of literature suggests that low-immunosuppression treatment regimens for IgAN focus on targeting and inhibiting the activation of B cells or CD38+ plasma cells, or promoting their exhaustion through the use of telitacicept. Given the positive efficacy and safety outcomes of telitacicept observed in the Phase II clinical trial for IgAN (7), we recommend discussing treatment options, including immunosuppressants and telitacicept, with the patient. Following a comprehensive discussion and the acquisition of informed consent, the patient commenced treatment with telitacicept in conjunction with optimized supportive care. Current evidence regarding the efficacy and safety of telitacicept in the IgAN population is predominantly derived from single case reports and case series. Shen et al. (15) reported a case in which treatment with an angiotensin II receptor blocker, corticosteroids, and mycophenolate mofetil did not result in significant improvement. However, upon the addition of telitacicept, the patient’s renal function improved and proteinuria decreased rapidly. A multicenter retrospective study involving 97 patients using telitacicept alone or in combination with steroid treatment confirmed that telitacicept can significantly and safely reduce proteinuria in patients with IgAN (16). Subsequent real-world studies further confirmed that telitacicept can result in a median reduction in urinary protein levels between 28.6% and 66.8%, with stable renal function (17, 18). In terms of efficacy, proteinuria in our case decreased from 2.8 g/day to 0.6 g/day after 18 months of treatment, representing a 78.6% reduction, which is consistent with the proteinuria-lowering efficacy of telitacicept reported in previous studies. In contrast, the patient in our case had a baseline eGFR of 26.95 mL/min/1.73 m², which improved to 35.69 mL/min/1.73 m^2^ after 18 months of treatment, indicating a gradual improvement in renal function. Notably, all published studies on telitacicept for IgAN have systematically excluded patients with CKD stage G4. This case represents the first reported successful treatment with telitacicept in an IgAN patient with such severe baseline renal impairment and a high degree of chronic pathological lesions, thereby addressing a critical evidence gap in existing research.

The rationale for excluding patients with CKD stage G4 from telitacicept clinical studies for IgAN is multifaceted. Primarily, from the clinical trial design perspective, novel drug trials tend to prioritize the enrollment of patients with relatively preserved renal function to ensure safety and maintain cohort homogeneity (7). The exclusion aims to minimize the complex confounding factors associated with renal impairment (such as potential drug accumulation risk and multiple complications), which could obscure the clear assessment of efficacy and safety. Furthermore, in accordance with global drug development and regulatory practices (19), novel therapeutic agents are initially evaluated in populations with a well-defined benefit-risk profile before extending indications to populations with complex comorbidities. This framework further supports the rationale for our clinical observation of telitacicept use in a patient with severe renal impairment. Additionally, telitacicept is not primarily renally cleared, which may theoretically support its use in patients with lower eGFR. The linear pharmacokinetic profile of telitacicept, characterized by dose-proportional exposure of both total and free drug within the 80–240 mg range, along with its relatively long terminal half-life in healthy subjects, provides a predictable pharmacological basis for determining and adjusting the treatment dose in our clinical practice (20). The current study exemplifies a personalized therapeutic approach and offers significant preliminary evidence for the potential future application of telitacicept in more extensive real-world studies or prospective investigations involving patients with advanced CKD.

Regarding the safety profile of telitacicept, published clinical trials and real-world studies consistently report a well-tolerated adverse event profile. The most frequently observed adverse events include upper respiratory tract infections, injection site reactions, urinary tract infections, and reversible reductions in circulating immunoglobulins (7, 16–18). These events are predominantly mild to moderate in severity, with a very low incidence of severe adverse events. In the present case, the patient was administered telitacicept for 18 months, during which no drug-related serious adverse events were observed, thereby further corroborating the safety of telitacicept.

The case report indicates a relatively safe therapeutic strategy for patients with IgAN and CKD stage G4 who exhibit active immune-mediated pathological lesions. Nevertheless, due to the absence of a control group and the limitation of being a single-patient case study, definitive conclusions regarding the treatment’s efficacy and safety in this population cannot be drawn. Telitacicept is currently undergoing Phase III clinical trials for the treatment of IgAN, during which its efficacy and safety will be further assessed.

In conclusion, this case shows that telitacicept can significantly decrease proteinuria, maintain kidney function, and postpone the advancement to ESRD, even when eGFR is below 30 mL/min/1.73 m², supporting its potential as a safe and effective treatment. This report indicates that additional research in this patient group could have clinical value.

## Data Availability

The original contributions presented in the study are included in the article/supplementary material. Further inquiries can be directed to the corresponding author.
